# Supplementary material for: A Humanized Yeast Phenomic Model of Deoxycytidine Kinase to Predict Genetic Buffering of Nucleoside Analog Cytotoxicity
Source: Genes (Basel). 2019 Sep 30;10(10):770. doi: 10.3390/genes10100770 (PMC6826991; doi:10.3390/genes10100770)
Supplement: Supplementary file 1 [file genes-10-00770-s001.zip › Additional_Files/Additional_File7_GOTermHeatmaps.zip!/Additional_File7_GOTermHeatmaps/mRNA_binding.pdf]

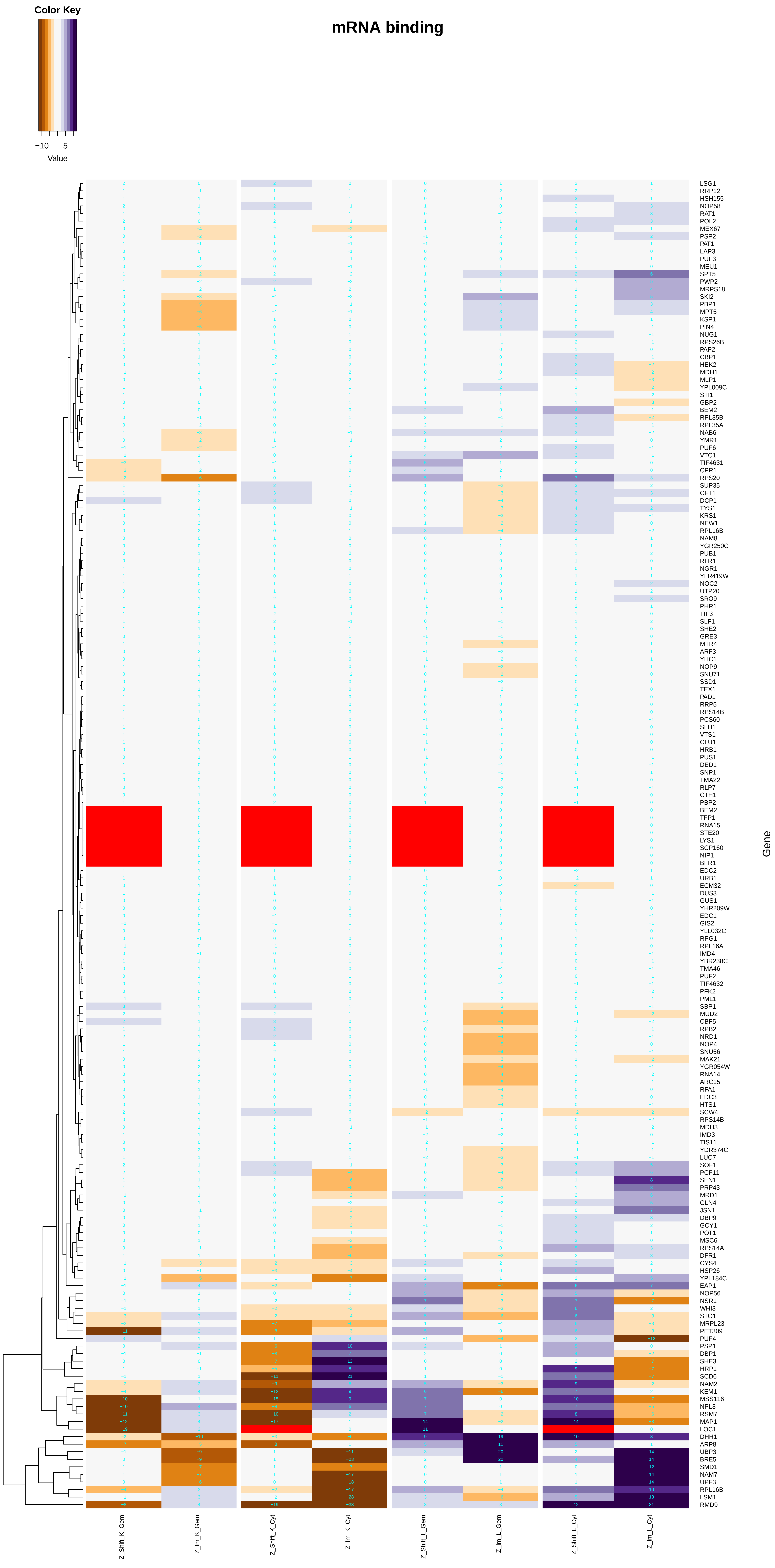
Color Key
−10
5
Value
mRNA binding
LSG1
RRP12
HSH155
NOP58
RAT1
POL2
MEX67
PSP2
PAT1
LAP3
PUF3
MEU1
SPT5
PWP2
MRPS18
SKI2
PBP1
MPT5
KSP1
PIN4
NUG1
RPS26B
PAP2
CBP1
HEK2
MDH1
MLP1
YPL009C
STI1
GBP2
BEM2
RPL35B
RPL35A
NAB6
YMR1
PUF6
VTC1
TIF4631
CPR1
RPS20
SUP35
CFT1
DCP1
TYS1
KRS1
NEW1
RPL16B
NAM8
YGR250C
PUB1
RLR1
NGR1
YLR419W
NOC2
UTP20
SRO9
PHR1
TIF3
SLF1
SHE2
GRE3
MTR4
ARF3
YHC1
NOP9
SNU71
SSD1
TEX1
PAD1
RRP5
RPS14B
PCS60
SLH1
VTS1
CLU1
HRB1
PUS1
DED1
SNP1
TMA22
RLP7
CTH1
PBP2
BEM2
TFP1
RNA15
STE20
LYS1
SCP160
NIP1
BFR1
EDC2
URB1
ECM32
DUS3
GUS1
YHR209W
EDC1
GIS2
YLL032C
RPG1
RPL16A
IMD4
YBR238C
TMA46
PUF2
TIF4632
PFK2
PML1
SBP1
MUD2
CBF5
RPB2
NRD1
NOP4
SNU56
MAK21
YGR054W
RNA14
ARC15
RFA1
EDC3
HTS1
SCW4
RPS14B
MDH3
IMD3
TIS11
YDR374C
LUC7
SOF1
PCF11
SEN1
PRP43
MRD1
GLN4
JSN1
DBP9
GCY1
POT1
MSC6
RPS14A
DFR1
CYS4
HSP26
YPL184C
EAP1
NOP56
NSR1
WHI3
STO1
MRPL23
PET309
PUF4
PSP1
DBP1
SHE3
HRP1
SCD6
NAM2
KEM1
MSS116
NPL3
RSM7
MAP1
LOC1
DHH1
ARP8
UBP3
BRE5
SMD1
NAM7
UPF3
RPL16B
LSM1
RMD9
Gene
Z_Shift_K_Gem
Z_Im_K_Gem
Z_Shift_K_Cyt
Z_Im_K_Cyt
Z_Shift_L_Gem
Z_Im_L_Gem
Z_Shift_L_Cyt
Z_Im_L_Cyt

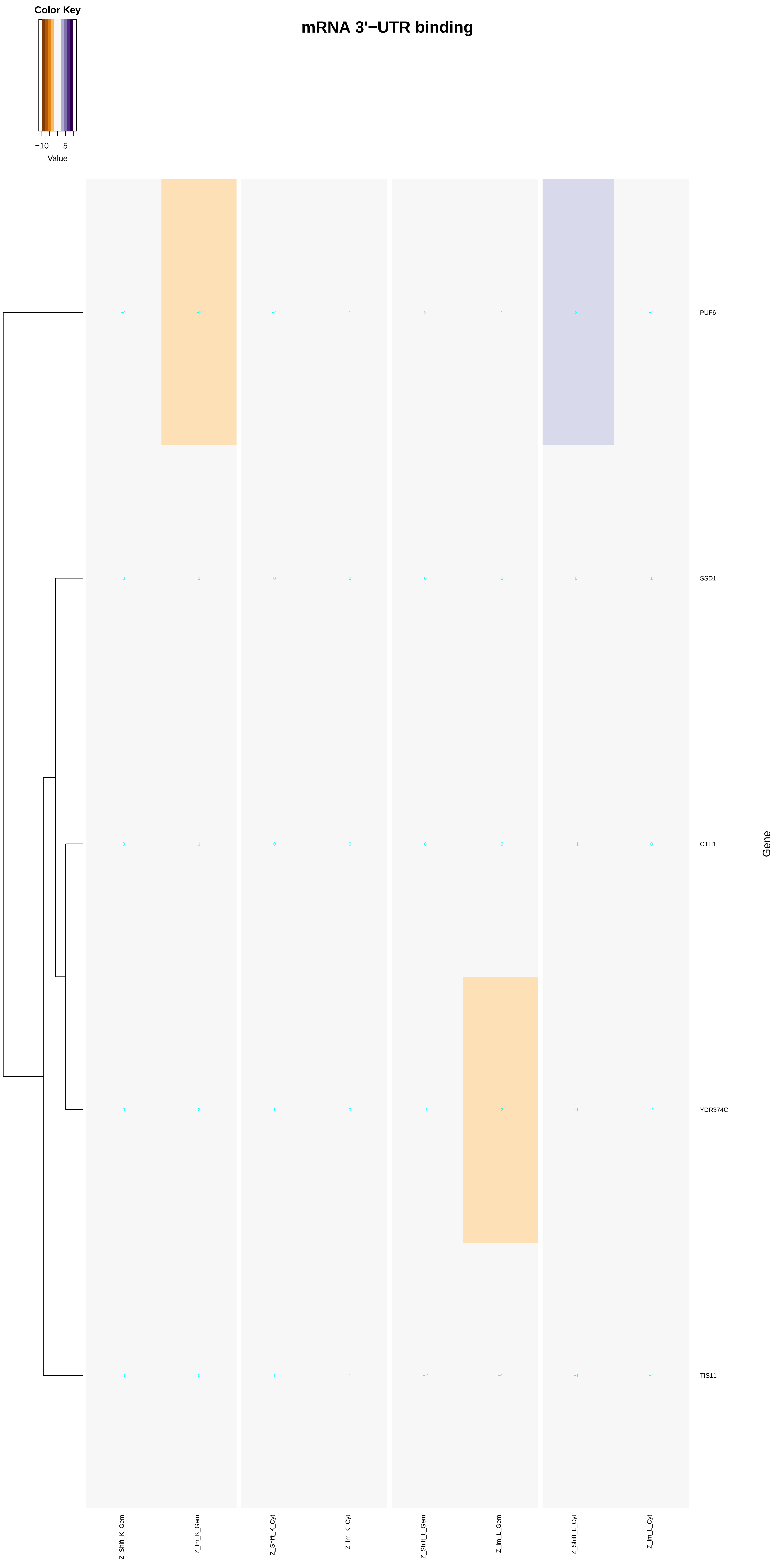

Color Key
−10
5
Value
mRNA 3'−UTR binding
PUF6
SSD1
CTH1
YDR374C
TIS11
Gene
Z_Shift_K_Gem
Z_Im_K_Gem
Z_Shift_K_Cyt
Z_Im_K_Cyt
Z_Shift_L_Gem
Z_Im_L_Gem
Z_Shift_L_Cyt
Z_Im_L_Cyt

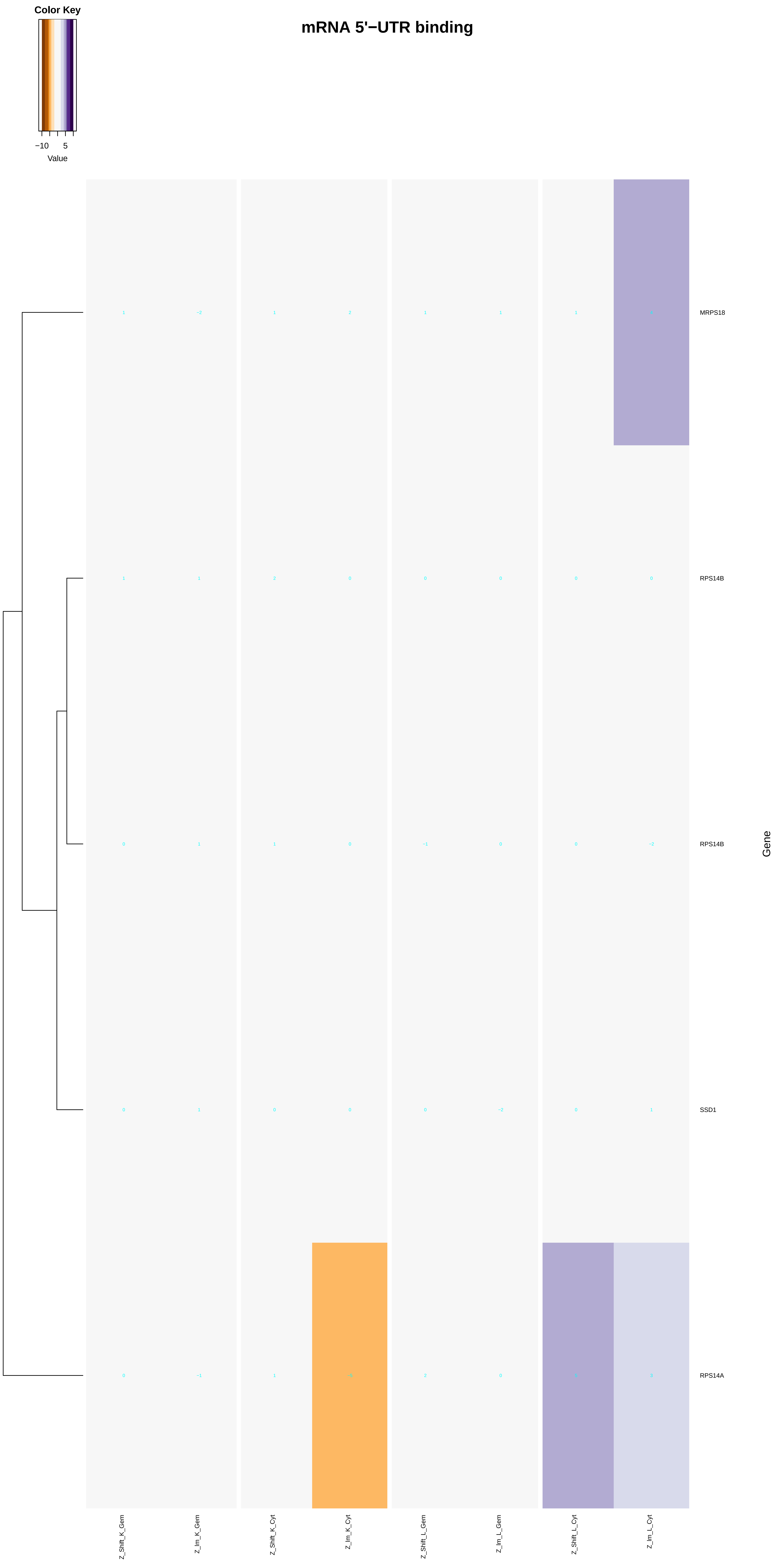
Color Key
−10
5
Value
mRNA 5'−UTR binding
MRPS18
RPS14B
RPS14B
SSD1
RPS14A
Gene
Z_Shift_K_Gem
Z_Im_K_Gem
Z_Shift_K_Cyt
Z_Im_K_Cyt
Z_Shift_L_Gem
Z_Im_L_Gem
Z_Shift_L_Cyt
Z_Im_L_Cyt
